# The Use of an MEG/fMRI-Compatible Finger Motion Sensor in Detecting Different Finger Actions

**DOI:** 10.3389/fbioe.2015.00205

**Published:** 2016-01-11

**Authors:** Xinyi Yong, Yasong Li, Carlo Menon

**Affiliations:** ^1^MENRVA Research Group, School of Engineering Science, Simon Fraser University, Burnaby, BC, Canada

**Keywords:** MEG/fMRI compatible, finger sensor, stroke, classification of finger actions, support vector machines

## Abstract

This paper explores the use of a novel device in detecting different finger actions among healthy individuals and individuals with stroke. The device is magnetoencephalography (MEG) and functional magnetic resonance imaging (fMRI) compatible. It was prototyped to have four air-filled chambers that are made of silicone elastomer, which contains low magnetizing materials. When an individual compresses the device with his/her fingers, each chamber experiences a change in pressure, which is detected by a pressure sensor. In a previous recent work, our device was shown to be MEG/fMRI compatible. In this study, our research effort focuses on using the device to detect different finger actions (e.g., grasping and pinching) in non-shielded rooms. This is achieved by applying a support vector machine to the sensor data collected from the device when participants are resting and executing the different finger actions. The total number of possible finger actions that can be executed using the device is 31. The healthy participants could perform all the 31 different finger actions and the average classification accuracy achieved is 95.53 ± 2.63%. The stroke participants could perform all the 31 different finger actions with their healthy hand and the average classification accuracy achieved is 83.13 ± 6.69%. Unfortunately, the functions of their affected hands are compromised due to stroke. Thus, the number of finger actions they could perform ranges from 2 to 24, depending on the level of impairments. The average classification accuracy for the affected hand is 83.99 ± 16.38%. The ability to identify different finger actions using the device can provide a mean to researchers to label the data automatically in MEG/fMRI studies. In addition, the sensor data acquired from the device provide sensorimotor-­related information, such as speed and force, when the device is compressed. Thus, brain activations can be correlated with this information during different finger actions. Finally, the device can be used to assess the recovery of the sensory and motor functions of individuals with stroke when paired with fMRI.

## Introduction

Devices that detect finger movements can be utilized in evaluating motor functions of human hands. Common solutions for detecting finger movements include piezoelectric buttons (Zatsiorsky et al., 1998, 2000), load cells (Edgren et al., 2004; Boonstra et al., 2005; Krejci et al., 2007), optical switches (De Luca et al., 2007), and gloves (Schaechter et al., 2006; Vanello et al., 2008), which are loaded with electronics, such as accelerometer and radio emitter. Buttons/straps functionalized by piezoelectric components (Zatsiorsky et al., 1998, 2000) and load cells (Edgren et al., 2004; Boonstra et al., 2005; Krejci et al., 2007) detect forces exerted by individual fingers. These devices keep finger positions stationary and measured the compression forces when the fingers flex toward the palm. Technically, these devices fall into the category of dynamometer, which measures the force rather than the movement of fingers. Optical switches (De Luca et al., 2007) and electronic buttons (Schwartz et al., 2010) provide information on whether a finger reaches a designated position. The data collected basically are binary, i.e., “on” or “off” of a finger reaching the predefined position. These switches provide few information of the motor function, as the fingers can activate passively, known as the enslaving effect. Gloves (Schaechter et al., 2006; Vanello et al., 2008) with electronics, on the other hand, can detect finger movements more precisely than the technologies discussed earlier. The 3D trajectories of finger movements can be recorded but not the force exerted during movements. The use of these gloves usually does not involve touching any other devices. Thus, the system provides no sensory information.

The correlation between the motor functions of human hands and brain activities have been of interest among neuroscientists for a long time (Volkmann et al., 1998; Fuchs et al., 2000; Jerbi et al., 2007; Waldert et al., 2008). There are a few devices for monitoring brain activities. Functional magnetic resonance imaging (fMRI) and magnetoencephalography (MEG) are widely used due to the fact that they are non-invasive and easy to set up (Lystad and Pollard, 2009). fMRI applies high magnetic field to the measuring participants (Ogawa et al., 1992), while MEG relies on highly sensitive sensors arrays to detect magnetic field difference during neuron firings (Hämäläinen et al., 1993). Collecting data from MEG/fMRI and finger movement sensors simultaneously placed a challenge since the signals from both devices interfere with each other (Tsekos et al., 2007). Most of the technologies developed for finger movement detection, as introduced previously, are manufactured by metals and contain electronic components. These materials can easily introduce noise to the highly sensitive sensors of MEG. Also, as these materials are movable under high magnetic field in an fMRI environment, the participant under measurement could be in danger. Therefore, the choices of material for the finger movement sensor are limited to low magnetizing and low ferromagnetic materials. Furthermore, the digitization of the finger movement signals are preferably placed outside the MEG/fMRI shielded environment. Some researchers (Yoo and Jolesz, 2002; Pilgramm et al., 2009) also use video cameras to observe finger movements when collecting neural activity data. While video cameras are commonly installed in MEG/fMRI shielded rooms as a measure of safety assurance, the recording only provides information of finger gestures but not force and sensory information of the movements. Besides, it is difficult to synchronize the video recording with the neural activity.

In this work, we use a novel polymeric device to detect finger movements of both healthy and poststroke participants. The device, also referred as finger sensor in our previous work (Li et al., submitted), is made of low magnetizing material and proven to have no interference with an MEG. As the signals collected from the finger sensor has a high signal-to-noise ratio (SNR), reliable detection of finger movements using a supported vector machine is made possible. Both healthy and stroke participants were involved in the tests for assessing the performance of the detection of finger movements. The sensor signals collected from the healthy and poststroke participants were also analyzed for further interpretation of the different accuracies achieved using the machine learning method. The promising results of finger pattern recognition enable future MEG/fMRI studies, which paired with the finger sensor to measure motor-related information of different finger actions. The remaining of the paper is organized as follows: material and methods are provided in Section “Materials and Methods”; results from both healthy and poststroke participants are presented in Section “Results”; and finally the discussion of the experimental results is given in Section “Discussion.”

## Materials and Methods

This section first provides details about the design of the finger sensor system. Then, the inclusion and exclusion criteria of the participants as well as the experimental procedures are presented. Finally, the machine-learning algorithm used for the classification of different finger actions and the metrics employed for performance evaluation are elaborated. All the methods within this study were in compliance with the declaration of Helsinki and were approved by the Simon Fraser University (SFU) Office of Research Ethics (#2012s0527). All the participants gave informed consent before taking part in the experiment.

### Finger Sensor System

**Figure 1:**
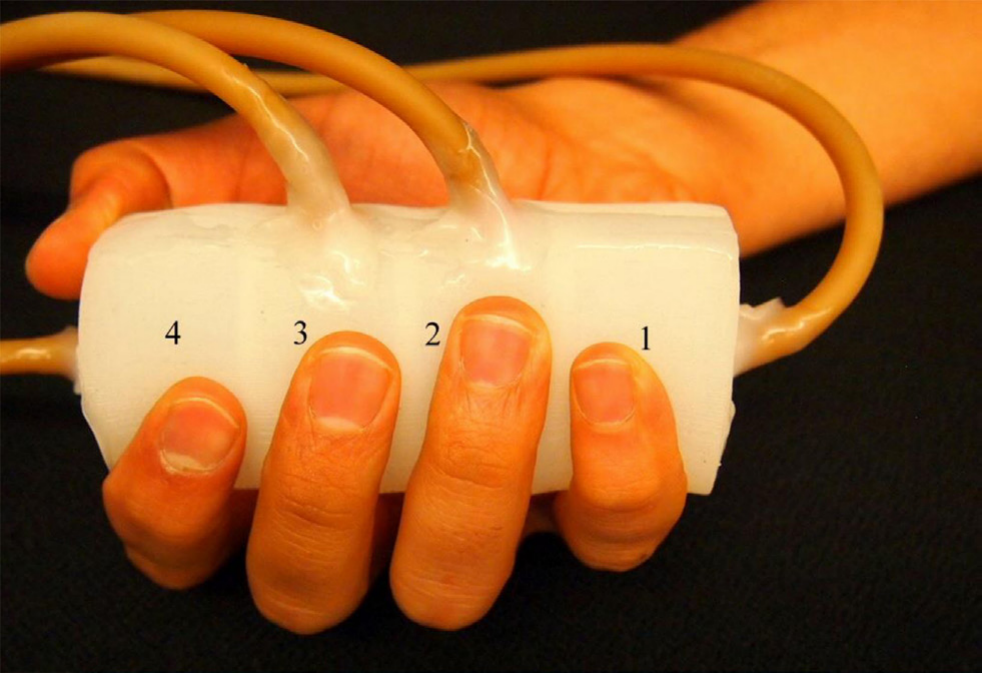
**Finger sensor**. The finger sensor consists of four chambers labeled as “1”–“4.” When holding the finger sensor, the thumb and index fingers are placed on chamber 4. The middle, ring, and little fingers are placed on chamber 3, 2, and 1, respectively.

The finger sensor is made of silicone elastomer (TC-5005, BJB enterprise) with a 3D-printed mold (Li et al., submitted). It consists of four chambers, which have the same size and are arranged in a line within a cylinder (see Figure 1). Each of the chambers is connected to a pressure sensor via a rubber tube. The pressure sensors (±25 kPa, MPXV7025, freescale semiconductor) measure the pressure changes of the chambers. The voltage outputs recorded from the four pressure sensors are sent to a bridge controller (PhidgetBridge 4-Input 1046, Phidgets, Inc.) that is connected to a computer via a USB cable. The raw signals are acquired at a sampling rate of 16 Hz in this study and saved to the computer for offline analysis.

The finger sensor can be held with a right or left hand. An example is demonstrated in Figure 1 where the finger sensor is held with a right hand. Each of the fingers (middle, ring, and little fingers) is placed on one of the chambers (labeled as “3,” “2,” and “1,” respectively). Both the thumb and index fingers share the chamber labeled as “4.” The finger sensor is designed to consist of only four chambers because when an individual grasps a cylindrical object, the thumb will naturally be placed opposite to the other four fingers. When one or more fingers compress the finger sensor, pressure changes will take place and result in changes in the raw signals (voltage outputs). It is also important not to place the fingers on the wall that separates the chambers so that the pressure change in each chamber can be traced back to the corresponding finger movements.

The voltage outputs can also provide information about the force exerted by the fingers (Li et al., submitted). The voltage is directly proportional to the chamber pressure (Freescale Semiconductor Inc., n.d.). Thus, the chamber pressure can be directly converted to the amount of force exerted by the corresponding finger. For example, a 0.1 V output from the pressure sensor indicates a chamber pressure of ~1.1 kPa. A finger (e.g., index, middle, or ring finger) of a female adult with the height of 1.55 m has roughly a surface contact area of 14 cm^2^ when it is compressing the cylinder. Therefore, the force exerted by the finger on the chamber is ~1.54 N. Also, the finger-moving velocity can be calculated using the following equation (Li et al., submitted):

$$F=m\frac{dv}{dt}$$

$$∴dv=\frac{Fdt}{m}$$

where *m* is the mass of the finger, *v* is velocity, *t* is time, and *F* is force. The mass of the finger can be estimated based on the close proximity between the human body average density and the water density at 1 kg/m^3^ (Krzywicki and Chinn, 1967).

### Participants

Five healthy individuals and five individuals with chronic stroke (>6 months after stroke) were recruited. The inclusion criteria for stroke participants include (a) age range from 35 to 85 years old, (b) poststroke duration more than 6 months, and (c) ability to give informed consent. Individuals with stroke who have other neurological conditions in addition to stroke and unstable cardiovascular diseases are excluded from the study. To assess the motor functions and impairment severity of the stroke participants, the Wolf motor function test (WMFT) (Wolf et al., 2001) and the upper-extremity subtest of the Fugl-Meyer (FM-UE) (Gladstone et al., 2002) were employed.

### Experimental Procedures

In the beginning of the experiment, each healthy participant was asked to hold the finger sensor with his/her dominant hand (without compressing the finger sensor with any of the fingers). Approximately 10 s worth of data were recorded and labeled as the baseline or rest data. Next, the participant was instructed to perform 31 different finger actions (i.e., compress the finger sensor with one or more fingers). Table 1 provides details about the finger(s) involved in the 31 different finger actions. For each finger action, the participant was asked to compress and hold for 5 s, followed by a 5-s rest interval. Each finger action was repeated for five times. The participant was free to decide the amount of force to apply to the finger sensor for each finger action.

**Table 1:** **Finger actions**.

Compress with one finger	Compress with two fingers	Compress with three fingers	Compress with four fingers	Compress with five fingers	Total number of classes
5 classes: F1, F2, F3, F4, F5	10 classes: F12, F13, F14, F15, F23, F24, F25, F34, F35, F45	10 classes: F123, F125, F125, F134, F135, F145, F234, F235, F245, F345	5 classes: F1234, F1235, F1245, F1345, F2345	1 class: F12345	31 classes

*All the finger actions that could be performed using the finger sensor*.

*F, finger; 1, thumb; 2, index finger; 3, middle finger; 4, ring finger; 5, little finger/pinky*.

The same experimental procedures described above were applied to the stroke participants except that each of them was asked to hold the finger sensor with his/her affected hand. Also, as stroke impairs the motor functions of the affected hand, the stroke participants were not able to perform all the 31 different finger actions. For three of the five stroke participants, the test was repeated with their healthy hands for comparison purposes.

### Classification of Finger Actions

The signals collected from the four pressure sensors of the finger sensor have a high SNR (Li et al., submitted). Thus, the features used for classification were the voltage output measured from each of the sensor, resulting in a total number of four features.

This is a multiclass classification problem. If the number of finger actions performed by the participant is *N*, then, the classifier will classify (*N* + 1) classes of data including the baseline or rest data. For example, the healthy participants completed all the 31 different finger actions and for each of them, a 32-class classifier was set up and evaluated.

In this study, a support vector machine (SVM) with a radial basis function (RBF) kernel (Bishop, 2006) was used to classify the multiclass data. Given some training data with labels, SVM solves the following optimization problem (Hsu et al., 2003):

$$\underset{w,\;b,\;\varepsilon }{\min}\;\frac{1}{2}w^{T}w\;+\;C\sum_{i=1}^{l}\xi_{i}$$

$$\text{subject to }y_{i}(w^{T}\phi (x_{i})+b)\geq 1\;-\;\xi_{i}$$

where *x*_i_ ∈ ℜ^4^ is the feature vector (i.e., the voltage output measured from each of the four chamber), *y_*i*_* is the corresponding label of *x_*i*_*, *i* is the index of the training sample, *w* is the normal vector to the hyperplane, *b* is the bias term, *C* > 0 is the penalty parameter of the error ξ*_*i*_*, and ϕ is the function that maps *x_*i*_* into a high-dimensional space. The kernel function, $K(x_{i},\;x_{j})=\;\phi {\left(x_{i}\right)}^{T}\phi (x_{j})$ for RBF is defined below (Hsu et al., 2003):

$$K(x_{i},\;x_{j})=e^{-\gamma \left|\right|x_{i}-x_{j}\left|\right|}{\;}^{{\;}^{2}},\;\gamma >0$$

where γ is the kernel parameter.

The data collected were split into three partitions: the first partition was used to train the classifier, the second partition was used for cross-validation, and the third partition was used to evaluate the performance of the classifier. The cross-validation data were important in the process of optimizing the two parameters of the SVM classifier, i.e., the kernel parameter gamma (γ) and the penalty weight (*C*). The optimal parameters were obtained from a grid search with γ ranging from 2^−15^ to 2^3^ and *C* ranging from 2^−5^ to 2^15^ (Hsu et al., 2003).

To apply SVM to a multiclass problem, a one-against-one strategy was employed. More specifically, *K*(*K* − 1)/2 binary classifiers for a *K*-class problem were trained. During testing, all these binary classifiers were applied to new and unseen samples. For each sample, the class that received the most number of votes won (Bishop, 2006).

The classification accuracy, which is the percentage of the correctly classified data, was used as a metric to assess the performance of a classifier. For each participant, both the cross-validation accuracy obtained from the second partitions of the data and the classification accuracy obtained from the third partitions of the data were computed.

## Results

### Healthy Individuals

The healthy participants, aged between 27 and 34 (31.6 ± 2.7), are right handed (see Table 2). They were able to perform all the 31 different finger actions.

**Table 2:** **Healthy participant information**.

Participant	Age	HH
P01	32	Right
P02	33	Right
P03	27	Right
P04	32	Right
P05	34	Right
Mean ± SD	31.6 ± 2.7	

*Age and handedness (HH) for each healthy participant*.

**Figure 2:**
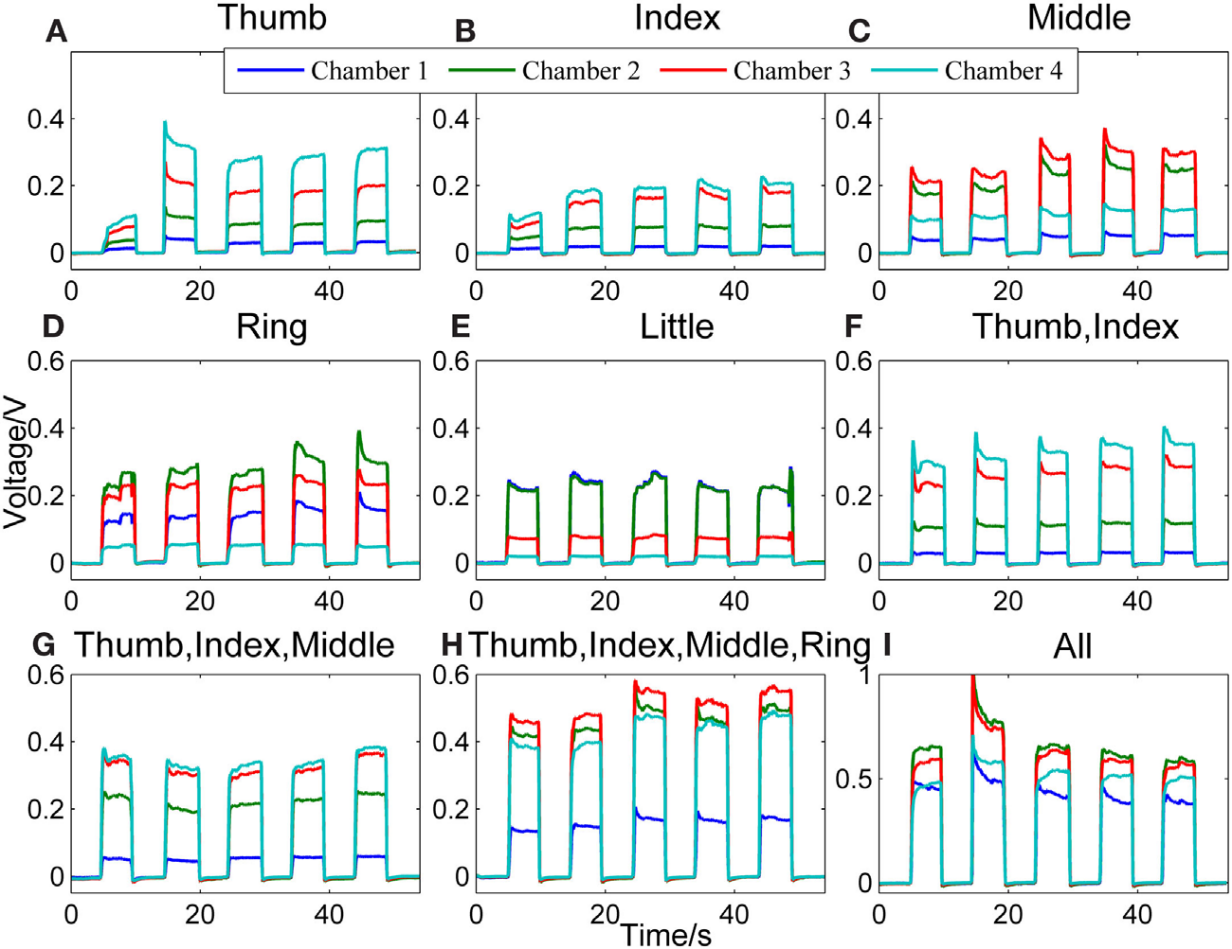
**Representative signal patterns for different finger actions**. Signals recorded from the four chambers of the finger sensor when nine different types of finger actions were performed, which include compressing the sensor body with **(A)** thumb, **(B)** index finger, **(C)** middle finger, **(D)** ring finger, **(E)** little finger, **(F)** thumb + index finger, **(G)** thumb + index + middle fingers, **(H)** thumb + index + middle + ring fingers, and **(I)** all fingers, respectively.

Figure 2 shows a representative data pattern corresponding to each of the following finger actions [the number(s) in the square brackets listed after the name of one or more fingers represents the chamber(s) being compressed]: (Figure 2A) thumb [4], (Figure 2B) index finger [4], (Figure 2C) middle finger [3], (Figure 2D) ring finger [2], (Figure 2E) little finger [1], (Figure 2F) thumb + index finger [1], (Figure 2G) thumb + index + middle fingers [4, 3], (Figure 2H) thumb + index + middle + ring fingers [4, 3, 2], and (Figure 2I) all fingers [4, 3, 2, 1]. The *y* axes represent the voltage measurements from the pressure sensors, and the *x* axes represent the time.

When the finger sensor is compressed by the thumb [4] (Figure 2A), index finger [4] (Figure 2B), or thumb and index fingers together (Figure 2F), the patterns of the signals are similar. This is due to the fact that both the thumb and index fingers are placed on the same chamber (i.e., chamber 4). Thus, when either or both of the fingers are in action, chamber 4 will experience the highest pressure, followed by chambers 3, 2, and 1. Despite of the similarity in terms of the order of the magnitude of the pressure in different chambers, the pressure difference between chambers 3 and 4 for these three different finger actions appears to be different. This difference may be attributed to the different locations where the thumb and index fingers are positioned.

For middle finger flexion [3] (Figure 2C), chamber 3 has the highest pressure, followed by chamber 2 (where the ring finger is placed). This could be due to the enslaving effect, i.e., the ring finger also produces some unintentional force even when the instructed finger is the middle finger (Wilhelm et al., 2014). The pressure of chamber 4, on the other hand, is the third highest. This is because the thumb and index fingers were holding the finger sensor body when the middle finger was flexing.

For the ring finger flexion [2] (Figure 2D), chamber 2 experiences the largest change in pressure since the ring finger is placed on the chamber. A relatively high pressure is observed in chambers 3 and 1, respectively because both the neighboring middle and little fingers also produces unintentional force due to enslaving when the ring finger moves. Chamber 4, where the thumb and index fingers are placed to hold the finger sensor body, has the lowest pressure among the four. The enslaving effect does not affect the thumb and index fingers when the ring finger flexes.

For the little finger flexion [1] (Figure 2E), chambers 1 and 2 experience high pressure, followed by chambers 3 and 4. The ring finger is apparently moving with the little finger when the little finger is compressing the chamber.

When the finger actions involve the thumb, index, and middle fingers [4, 3] (Figure 2G), the signal patterns are similar with that of the thumb and/or index fingers [4] (Figures 2A,B,F). However, the pressure difference between chambers 3 and 4 is smaller in the case of Figure 2G. This is because the middle finger, which is placed on chamber 3, is also in action.

Next, Figure 2H shows the signal patterns obtained when all fingers except the little finger [4, 3, 2] applied force to compress the finger sensor. Chambers 2, 3, and 4 have high voltage outputs. Chamber 1, where the little finger is positioned, has the smallest change in pressure because the little finger produced the least force.

When the finger sensor body is compressed by all fingers [4, 3, 2, and 1] (Figure 2I), not all fingers apply the same force to the device. In this example, the ring and middle fingers produced more force. Thus, chambers 2 and 3 have a higher pressure than the other two chambers. The magnitude of the voltage (e.g., ~0.70 V in chamber 2) acquired from each chamber is on average larger than those when fewer fingers are involved in the finger actions. This result suggests that the grip force is much larger than the force produced by other finger actions.

Next, the performance of the classifier in classifying rest against the 31 finger actions is presented. As described earlier, the data were divided into three parts: part 1 for training the classifier, part 2 for cross-validation, and part 3 for testing or evaluating the performance of the classifier on unseen samples. Table 3 presents both the cross-validation and testing accuracies obtained from the five healthy participants, P01-P05. On average, the classifier achieved 98.74 ± 1.79% on cross-validation data and 95.53 ± 2.63% on testing data. The results are encouraging as the classifier is able to discriminate all the different finger actions despite of the similarities in their associated data patterns in some actions (e.g., thumb, index finger, thumb + index fingers). We observe that most errors or misclassifications correspond to finger actions that involved the compression of the same chambers. For example, some samples of the action involving the index + middle + ring fingers [4, 3, 2] were misclassified as that involving the thumb + index + middle + ring fingers [4, 3, 2].

**Table 3:** **Classification performance for healthy participants**.

Participant	Cross-validation accuracy (%)	Test accuracy (%)
P01	99.03	93.38
P02	95.58	92.30
P03	99.85	96.43
P04	99.68	96.79
P05	99.54	98.74
Mean ± SD	98.74 ± 1.79	95.53 ± 2.63

*Classification accuracy achieved when an SVM was used to classify the signals acquired from the finger sensor when different finger actions were executed*.

### Individual with Stroke

Table 4 presents the demographics and stroke-related information of the five stroke participants. The participants, aged between 58 and 79 years (67.8 ± 7.7), are right handed. The affected hand for the participants is left (except S01). The WMFT time for the affected hand ranges from 1.0 to 83.6 s, with larger values indicating lower functioning levels. Next, the FM-UE scores for the affected hand range from 12 to 49, indicating mild to severe motor impairments. The maximum score for FM-UE is 66, and smaller FM-UM scores indicate more severe motor impairments.

**Table 4:** **Stroke participant information**.

Participant	Age	DAS (months)	HH	AH	WMFT	FM-UE
S01	65	115	Right	Right	83.6	12
S02	70	31	Right	Left	7.4	38
S03	58	34	Right	Left	66.5	26
S04	67	90	Right	Left	2.5	49
S05	79	27	Right	Left	1.0	38
Mean ± SD	67.8 ± 7.7	59.4 ± 40.4			32.2 ± 39.7	32.6 ± 24.1

*Age, duration after stroke (DAS), handedness (HH), affected hand (AH), WMFT scores, and FM-UE scores for each participant with stroke*.

*DAS, duration after stroke; HH, handedness; AH, affected hand*.

When asked to perform different finger actions with the affected hand, all the stroke participants were not able to complete all. The number of finger actions that was completed by S01–S05 is 2, 24, 8, 9, and 9, respectively. Three of the stroke participants (S03–S05) also completed the same procedures with their healthy hand. They successfully completed all the finger actions with their healthy hands.

**Figure 3:**
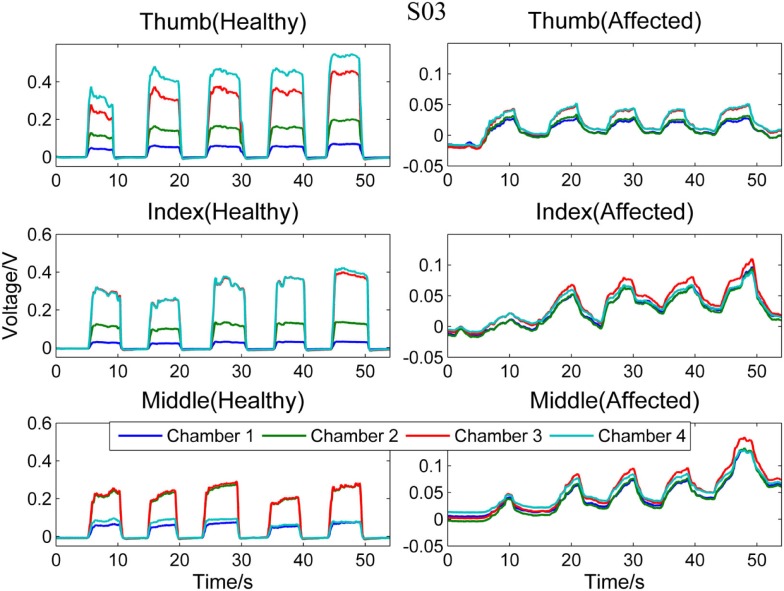
**Comparison between healthy and affected hands of S03**. Signals recorded from the four chambers of the finger sensor when three different types of finger actions were performed by S03, namely compressing the sensor body with thumb, index finger, and middle finger, respectively.

Figure 3 compares the signal patterns obtained when S03 was performing different single finger action [thumb [4], index [4], or middle finger [3]] with his healthy (left column) and affected (right column) hands. As shown in the figure, the pressure measurements for the affected hand are significantly smaller than the measurements for the healthy hand. Also, as S03 experienced difficulty on releasing his finger (affected hand) from pressing the finger sensor, the signals collected from the affected hand did not fully return to their baseline values before the next finger action. In contrast, the finger of S03’s healthy hand can press and release the finger sensor with a much higher degree of control. Nevertheless, S03’s healthy hand experiences a higher degree of enslaving effect compared to healthy participants when performing index or middle finger flexion (see Figure 3, left column). For example, when he pressed the finger sensor with his index finger [4], his middle finger [3] produced almost as much force as his index finger (middle plot of the left column in Figure 3); when he pressed with his middle finger, both chambers 2 and 3 registered high pressure changes (lower plot of the left column in Figure 3). The enslaving effect is even greater in his affected hand, especially when he was trying to perform index and middle fingers independently.

**Figure 4:**
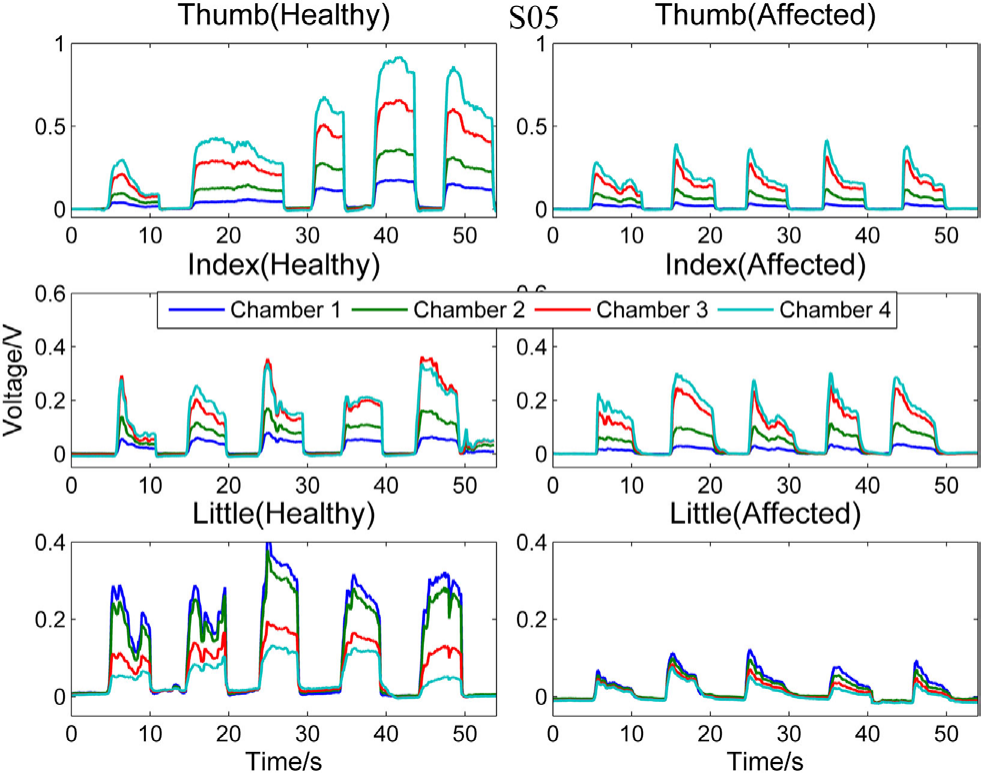
**Comparison between healthy and affected hands of S05**. Signals recorded from the four chambers of the finger sensor when three different types of finger actions were performed by S05, namely compressing the sensor body with thumb, index finger, and little finger, respectively.

Next, Figure 4 compares the signal patterns obtained from the healthy and affected hands when S05 performed single finger actions. S05 has better functioning levels compared to S03. Thus, the signal patterns obtained from both the healthy and affected hands are similar, except that the force production of the affected hand was smaller.

**Figure 5:**
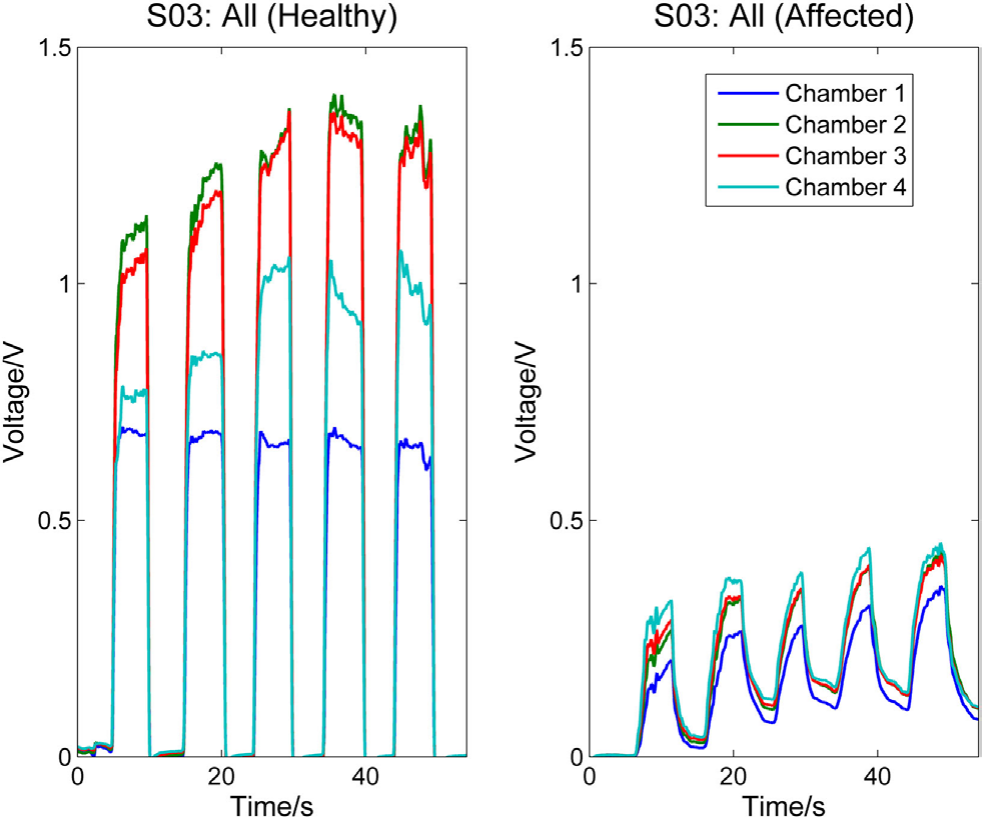
**Comparison between healthy and affected hands of S03 (grasp)**. Signals recorded from the four chambers of the finger sensor when S03 compressed it with all fingers (both healthy and affected).

**Figure 6:**
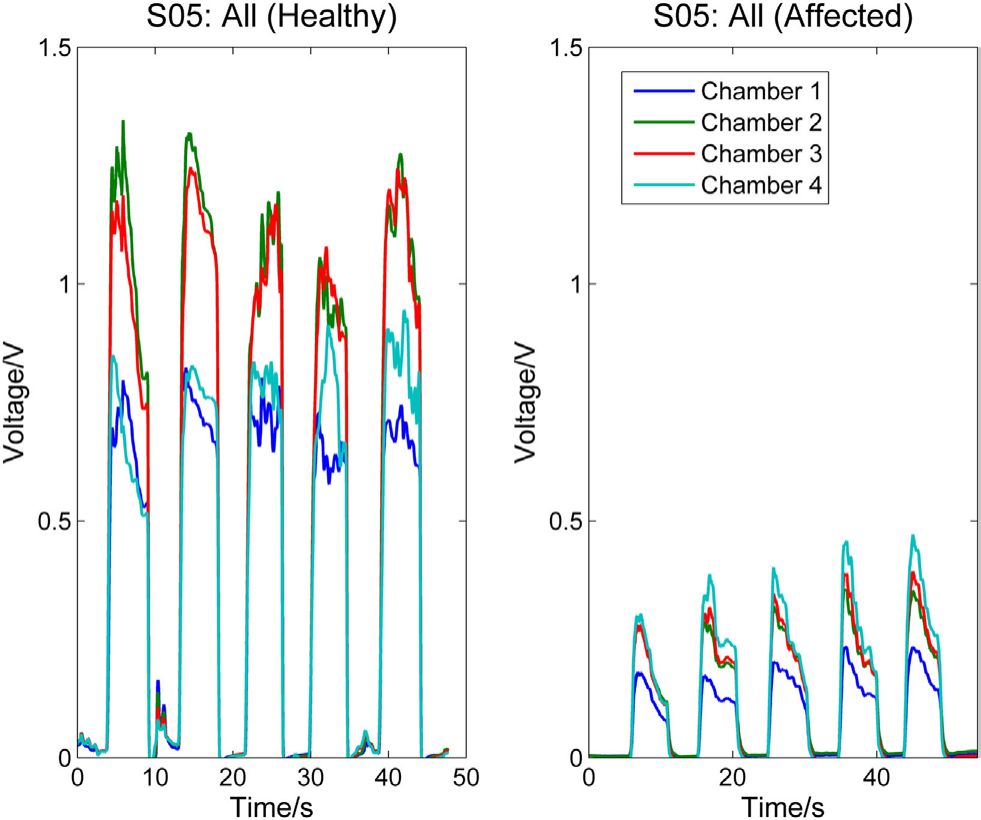
**Comparison between healthy and affected hands of S05 (grasp)**. Signals recorded from the four chambers of the finger sensor when S05 compressed it with all fingers (both healthy and affected).

Figures 5 and 6, respectively, compare the signal patterns obtained from both the healthy and affected hands when S03 and S05 were grasping the finger sensor with all the fingers. Similar to Figures 3 and 4, the force produced by the affected hand is smaller due to smaller voltage values recorded from the pressure chamber. When grasping with the healthy hand, the middle [3] and ring fingers [2] applied larger force. In contrast, when grasping with his affected hand, the first three digits (the thumb, index, and middle fingers) of both S03 and S05 appear to have more strength.

Next, the performance of the classifier in classifying rest against the different finger actions is presented. Table 5 presents both the cross-validation and testing accuracies obtained from the healthy hand of the stroke participants, S03–S05. On average, the classifier achieved 94.20 ± 4.57% on cross-validation data and 83.13 ± 6.69% on testing data. Table 6 reports the cross-validation and testing accuracies obtained from the affected hand of all the stroke participants, S01–S05. The number of classes and the different finger actions that were able to be performed by each participant are also listed in the table. The classifier achieved an average accuracy of 96.07 ± 6.07% on cross-validation data and 83.99 ± 16.38% on testing data. S01, who has the most severe level of impairments, could only perform grasp, but no other finger actions. The binary classification of rest and grasp led to high classification accuracy of 100.0% on both the cross-validation and testing data. S02, on the other hand, was able to perform the most number of finger actions (24). The classification accuracy achieved when tested on new unseen testing data was 60.21%. The low classification accuracy could be due to the confusion caused by enslaving effects. For example, the classifier misclassified some samples of middle finger [3] movement to the finger action involving index [4], middle [3], and ring fingers [2].

**Table 5:** **Classification performance for participants with stroke (healthy hand)**.

Participant	Cross-validation accuracy (%)	Test accuracy (%)
S03	97.86	90.82
S04	89.07	79.94
S05	95.66	78.64
Mean ± SD	94.20 ± 4.57	83.13 ± 6.69

*Classification accuracy achieved when an SVM was used to classify the signals acquired from the finger sensor when different finger actions were executed*.

**Table 6:** **Classification performance for participants with stroke (affected hand)**.

Participant	Number of classes	Cross-validation accuracy (%)	Test accuracy (%)
S01	2 (rest, f12345)	100.0	100.0
S02	24 (rest, f1, f2, f3, f4, f5, f12, f13, f14, f15, f23, f25, f34, f35, f45, f123, f125, f134, f145, f234, f235, f245, f345, f12345)	85.63	60.21
S03	8 (rest, f1, f2, f3, f12, f123, f1234, f12345)	100.0	90.93
S04	9 (rest, f1, f2, f3, f4, f5, f12, f123, f12345)	95.93	74.35
S05	9 (rest, f1, f2, f3, f12, f15, f25, f2345, f12345)	98.82	94.47
Mean ± SD	10.40 ± 8.14	96.07 ± 6.07	83.99 ± 16.38

*Classification accuracy achieved when an SVM was used to classify the signals acquired from the finger sensor when different finger actions were executed. The finger actions that were executed by each participant were listed in the second column*.

## Discussion

In our previous work (Li et al., submitted), we have proposed a novel device that is made of silicone elastomer, which consists of low ferromagnetic materials. This device or finger sensor was designed to be MEG/fMRI-compatible so that it can be used in MEG and fMRI studies to investigate brain activations and sensorimotor functions of healthy individuals. The device can also be used to assess the recovery of the sensory and motor functions of individuals with stroke when paired with MEG/fMRI. The device’s property of being MEG compatible was validated.

In this paper, our research effort focuses on exploring the different finger actions that can be performed by both healthy individuals and individuals with stroke in non-shielded rooms. Besides, we also look into the feasibility of classifying all the different types of finger actions based on the signals recorded from the finger sensor. The ability to classify different finger actions can benefit MEG/fMRI studies that use the finger sensor to detect movements. First, automated labeling of the data achieved via the classification of finger actions can reduce labor-intensive work in labeling data in various MEG/fMRI studies. For example, comparing the brain activations when the fingers are voluntarily moved in a predefined (i.e., determined by the investigator) and random (determined by the participant) sequence. Furthermore, the device can be used to study finger interdependence and the enslaving effects due to different finger actions.

The finger sensor consists of four air-filled chambers that are made of silicone elastomer. When an individual compresses the device with his/her finger(s), each chamber experiences a change in pressure, which is detected by a pressure sensor. The signals recorded provide sensorimotor-related information, such as speed and force, when the device is compressed. The data can potentially be used to assess the dexterity of the hands. It is known that the dexterity of the affected hand of individuals with stroke, as evidenced by a decrease in the force, a reduction in the independence of finger movements, an increase in the timing, or an increase in the deficiency of finger sequencing (Térémetz et al., 2015). By comparing the signals recorded from both the healthy and affected hands of the stroke participants (e.g., Figures 3–6), it can be observed that the affected hand produced less force, i.e., it has less strength due to stroke. If the participants were asked to compress the device with maximal force using the healthy and affected hands, respectively, the strength difference between the two hands can be computed. Besides, the affected hand usually experienced fatigue (i.e., a drop in the voltage after achieving the peak) earlier than the healthy hand (e.g., Figure 6). Some individuals may also experience a decrease in the speed of returning the fingers to certain positions. For example, one of the participants, S03 experienced a decrease in the speed of pressing and releasing the device, as evidenced by Figures 3 and 5. Finally, from Figures 3–6, the effects of stroke on the independence of finger movements and coordination of fingers can also be observed.

The signals recorded from the finger sensor have a high SNR. Finger actions that activate different chamber(s) produce distinct signal patterns, which facilitate the classification of different finger actions. The number of possible finger actions that can be executed using the finger sensor is 31, which involves the flexion of different digits: the thumb, index, middle, ring, and little fingers. The healthy participants were able to perform all the 31 different finger actions and high average classification accuracies were achieved (see Table 3). The stroke participants could also perform all the 31 different finger actions with their healthy hand; the classifier can achieve a slightly lower accuracy (see Tables 5 and 6). As their affected hand’s dexterity was compromised due to stroke, the stroke participants have difficulty moving certain fingers independently and thus performing many of the finger actions instructed. The number of finger actions they can perform with their affected hand ranges from 2 to 24. For example, S01, who has the highest level of impairments, was only able to perform grasping using the device. No significant correlation exits between the number of finger actions the stroke participants can perform and their WMFT (*r* = −0.59, *p* = 0.29) and FM-UE scores (*r* = 0.50, *p* = 0.39), respectively. This may be caused by other confounding factors that also affect the number of finger actions an individual with stroke can perform, e.g., how motivated the person was when performing the different finger actions and whether or not the person had other problems, such as arthritis in joints. Participant S04, who has the largest FM score and second lowest WMFT score, could only perform eight finger actions because he was not able to move most of his fingers independently. This number of finger actions which can be performed is fewer than S02, who has a higher level of impairments compared to S04. The average accuracy obtained when classifying the different finger actions performed with the affected hand is 83.199 ± 16.38%.

One limitation of the finger sensor is that it is only suitable for studies involving finger flexion but not finger extension, abduction, or adduction. Also, the locations of the finger may shift from test to test when the finger sensor is in use. This limitation, however, can be overcome by attaching extra straps to fix the locations where the fingers compress.

In our future work, MEG studies will be conducted to further understand the neural representations and activities in the brain when different single/multi-finger actions are executed randomly or in a predefined sequence. The studies will involve both healthy individuals and individuals with neurological disorders, such as stroke. The signal patterns of different finger actions will be classified to automatically label the data for data analysis. Besides, correlation between brain activations and motor-related information, such as force and velocity, will be explored. The finger sensor can also potentially be used to study finger interdependence and enslaving effects on brain activities. Other metrics that can be potentially derived using the finger sensor include (i) the time taken to reach a certain percentage of the maximum voltage when a finger action is executed to assess neural response time, (ii) the decrease in the maximum voltage to assess the fatigue level of the fingers, and (iii) the drift in the baseline to assess how fast one can release the fingers after an action and the ability of an individual to relax after an action. These metrics are potentially useful in examining the effects of rehabilitation sessions on the recovering of finger functions.

## Author Contributions

XY and CM conceived the experiment; XY and YL designed and conducted the experiment; XY analyzed the data; XY and YL interpreted the results; XY, YL, and CM cowrote the paper.

## Conflict of Interest Statement

The authors declare that the research was conducted in the absence of any commercial or financial relationships that could be construed as a potential conflict of interest.

## References

[B1] Bishop C. (2006). in Pattern Recognition and Machine Learning, 1st Edn, eds Jordan M. Kleinberg J. Scholkopf B. (Springer). Available at: http://cache.freescale.com/files/sensors/doc/data_sheet/MPXV7025.pdf

[B2] Boonstra T. W. Clairbois H. E. Daffertshofer A. Verbunt J. van Dijk B. W. Beek P. J. (2005). MEG-compatible force sensor. J. Neurosci. Methods 144, 193–196. 10.1016/j.jneumeth.2004.11.004 15910977

[B3] De Luca C. Comani S. Di Donato L. Caulo M. Bertollo M. Luca Romani G. (2007). A-magnetic optic-mechanical device to quantify finger kinematics for fMRI studies of bimanual coordination. Brain Topogr. 19, 155–160. 10.1007/s10548-007-0022-5 17605100

[B4] Edgren C. S. Radwinx R. G. Irwin C. B. (2004). Grip force vectors for varying handle diameters and hand sizes. Hum. Factors 46, 244–251. 10.1518/hfes.46.2.244.37337 15359674

[B5] Freescale Semiconductor Inc. (n.d.). Freescale Semiconductor Inc., Integrated Silicon Pressure Sensor MPXV7025 Series Data Sheet, Rev.6.

[B6] Fuchs A. Jirsa V. K. Scott Kelso J. A. (2000). Theory of the relation between human brain activity (MEG) and hand movements. Neuroimage 11, 359–369. 10.1006/nimg.1999.0532 10806021

[B7] Gladstone D. J. Danells C. J. Black S. E. (2002). The Fugl-Meyer assessment of motor recovery after stroke: a critical review of its measurement properties. Neurorehabil. Neural Repair 16, 232–240. 10.1177/154596802401105171 12234086

[B8] Hämäläinen M. Hari R. Ilmoniemi R. J. Knuutila J. Lounasmaa O. V. (1993). Magnetoencephalography – theory, instrumentation, and applications to noninvasive studies of the working human brain. Rev. Mod. Phys. 65, 413–497. 10.1103/RevModPhys.65.413

[B9] Hsu C.-W. Chang C.-C. Lin C.-J. (2003). A Practical Guide to Support Vector Classification. Technical report, National Taiwan University Available at: http://www.csie.ntu.edu.tw/~cjlin/papers/guide/guide.pdf

[B10] Jerbi K. Lachaux J. P. N’Diaye K. Pantazis D. Leahy R. M. Garnero L. (2007). Coherent neural representation of hand speed in humans revealed by MEG imaging. Proc. Natl. Acad. Sci. U.S.A. 104, 7676–7681. 10.1073/pnas.0609632104 17442753 PMC1863498

[B11] Krejci E. Pinkus B. Trinque J. Michaud J. (2007). MRI/MEG Compatible Grip Force Dynamometer for Stroke Patients. Northeastern University Available at: http://hdl.handle.net/2047/d20000834

[B12] Krzywicki H. J. Chinn K. S. K. (1967). Human body density and fat of an adult male population as measured by water displacement. Am. J. Clin. Nutr. 20, 305–310. 6022006 10.1093/ajcn/20.4.305

[B13] Lystad R. P. Pollard H. (2009). Functional neuroimaging: a brief overview and feasibility for use in chiropractic research. J. Can. Chiropr. Assoc. 53, 59–72. 19421353 PMC2652631

[B14] Ogawa S. Tank D. W. Menon R. Ellermann J. M. Kim S. G. Merkle H. (1992). Intrinsic signal changes accompanying sensory stimulation: functional brain mapping with magnetic resonance imaging. Proc. Natl. Acad. Sci. U.S.A. 89, 5951–5955. 10.1073/pnas.89.13.5951 1631079 PMC402116

[B15] Pilgramm S. Lorey B. Stark R. Munzert J. Zentgraf K. (2009). The role of own-body representations in action observation: a functional MRI study. Neuroreport 20, 997–1001. 10.1097/WNR.0b013e32832d21fc 19579267

[B16] Schaechter J. D. Stokes C. Connell B. D. Perdue K. Bonmassar G. (2006). Finger motion sensors for fMRI motor studies. Neuroimage 31, 1549–1559. 10.1016/j.neuroimage.2006.02.029 16624582

[B17] Schwartz E. S. Edgar J. C. Gaetz W. C. Roberts T. P. (2010). Magnetoencephalography. Pediatr. Radiol. 40, 50–58. 10.1007/s00247-009-1451-y 19937237

[B18] Térémetz M. Colle F. Hamdoun S. Maier M. A. Lindberg P. G. (2015). A novel method for the quantification of key components of manual dexterity after stroke. J. Neuroeng. Rehabil. 12, 64. 10.1186/s12984-015-0054-0 26233571 PMC4522286

[B19] Tsekos N. V. Khanicheh A. Christoforou E. Mavroidis C. (2007). Magnetic resonance-compatible robotic and mechatronics systems for image-guided interventions and rehabilitation: a review study. Annu. Rev. Biomed. Eng. 9, 351–387. 10.1146/annurev.bioeng.9.121806.160642 17439358

[B20] Vanello N. Hartwig V. Tesconi M. Ricciardi E. Tognetti A. Zupone G. (2008). Sensing glove for brain studies: design and assessment of its compatibility for fMRI with a robust test. IEEE/ASME Trans. Mechatron. 13, 345–354. 10.1109/TMECH.2008.924115

[B21] Volkmann J. Schnitzler A. Witte O. W. Freund H. (1998). Handedness and asymmetry of hand representation in human motor cortex. J. Neurophysiol. 79, 2149–2154. 9535974 10.1152/jn.1998.79.4.2149

[B22] Waldert S. Preissl H. Demandt E. Braun C. Birbaumer N. Aertsen A. (2008). Hand movement direction decoded from MEG and EEG. J. Neurosci. 28, 1000–1008. 10.1523/JNEUROSCI.5171-07.2008 18216207 PMC6671004

[B23] Wilhelm L. A. Martin J. R. Latash M. L. Zatsiorsky V. M. (2014). Finger enslaving in the dominant and non-dominant hand. Hum. Mov. Sci. 33, 185–193. 10.1016/j.humov.2013.10.001 24360253 PMC3976954

[B24] Wolf S. L. Catlin P. A. Ellis M. Archer A. L. Morgan B. Piacentino A. (2001). Assessing Wolf motor function test as outcome measure for research in patients after stroke. Stroke 32, 1635–1639. 10.1161/01.STR.32.7.1635 11441212

[B25] Yoo S. S. Jolesz F. A. (2002). Functional MRI for neurofeedback: feasibility study on a hand motor task. Neuroreport 13, 1377–1381. 10.1097/00001756-200208070-00005 12167756

[B26] Zatsiorsky V. M. Li Z. M. Latash M. L. (1998). Coordinated force production in multi-finger tasks: finger interaction and neural network modeling. Biol. Cybern. 79, 139–150. 10.1007/s004220050466 9791934

[B27] Zatsiorsky V. M. Li Z. M. Latash M. L. (2000). Enslaving effects in multi-­finger force production. Exp. Brain Res. 131, 187–195. 10.1007/s002219900261 10766271

